# Chitosan Particles Complexed with CA5-HIF-1α Plasmids Increase Angiogenesis and Improve Wound Healing

**DOI:** 10.3390/ijms241814095

**Published:** 2023-09-14

**Authors:** Louis J. Born, Sameer Bengali, Angela Ting Wei Hsu, Sanaz Nourmohammadi Abadchi, Kai-Hua Chang, Frank Lay, Aerielle Matsangos, Christopher Johnson, Steven M. Jay, John W. Harmon

**Affiliations:** 1 Fischell Department of Bioengineering, University of Maryland, College Park, MD 20742, USA; 2 Hendrix Burn and Wound Healing Laboratory, Department of Surgery, Johns Hopkins University School of Medicine, Baltimore, MD 21205, USA; 3 Program in Molecular and Cell Biology, University of Maryland, College Park, MD 20742, USA

**Keywords:** nanoparticles, wound healing, non-viral gene therapy, hypoxia-inducible factor-1α

## Abstract

Wound therapies involving gene delivery to the skin have significant potential due to the advantage and ease of local treatment. However, choosing the appropriate vector to enable successful gene expression while also ensuring that the treatment’s immediate material components are conducive to healing itself is critical. In this study, we utilized a particulate formulation of the polymer chitosan (chitosan particles, CPs) as a non-viral vector for the delivery of a plasmid encoding human CA5-HIF-1α, a degradation resistant form of HIF-1α, to enhance wound healing. We also compared the angiogenic potential of our treatment (HIF/CPs) to that of chitosan particles containing only the plasmid backbone (bb/CPs) and the chitosan particle vector alone (CPs). Our results indicate that chitosan particles exert angiogenic effects that are enhanced with the human CA5-HIF-1α-encoded plasmid. Moreover, HIF/CPs enhanced wound healing in diabetic db/db mice (*p* < 0.01), and healed tissue was found to contain a significantly increased number of blood vessels compared to bb/CPs (*p* < 0.01), CPs (*p* < 0.05) and no-treatment groups (*p* < 0.01). Thus, this study represents a method of gene delivery to the skin that utilizes an inherently pro-wound-healing polymer as a vector for plasmid DNA that has broad application for the expression of other therapeutic genes.

## 1. Introduction

In the United States alone, non-healing wounds resulting from disease, age, or surgical intervention affect over 6.5 million people and cost over USD 10 billion to treat each year [1,2]. With an increasingly aging population and continual rise in diabetes mellitus, especially in the United States [3], chronic wounds are expected to rise. Currently, a variety of dressings exist for the management of complex wounds, ranging from polymer-based fibrous dressings to topical creams [4]. Other approaches that have been gaining traction involve loading primary dressings with active ingredients such as anti-microbial ions [5], pro-angiogenic peptides [6], extracellular vesicles [7], and even viable cells [8]. However, none of these has emerged as the consensus “gold standard” for wound care, suggesting the need to develop new approaches for wound healing.

Wound healing can be classified into four stages—hemostasis, inflammation, proliferation, and maturation—and gene expression during each stage and between transitions is extremely dynamic, with appropriate levels of specific genes being critical for repair and regeneration [9]. Dysregulation of key genes can impair healing [10,11,12,13], and a recent clinical trial has been focused on identifying differences in gene expression profiles between non-healing and normal-healing wounds [14]. One master regulator of angiogenesis, a critical process in wound healing, is hypoxia-inducible factor-1α (HIF-1α). HIF-1α has been shown to be a significant component for appropriate wound healing in a number of studies utilizing chronic wound-healing models [15,16,17,18] and other revascularization strategies [19,20]. Prior work from our group has shown that a reduction in HIF-1α expression in elderly and diabetic mice causes impairments in wound healing [21,22,23]. Additionally, we have shown that local, injectable delivery of a plasmid expressing a mutant form of human HIF-1α (CA5-HIF-1α), in which amino acids 392–520 are deleted to prevent O2-dependent degradation by prolyl hydroxylases [24], can successfully improve skin flap survival in a rat ischemic pedicle flap model [25].

Thus, delivery of HIF-1α (as well as other genes) has been widely explored in wound therapy. Initial efforts for the delivery of DNA plasmids focused on viral vectors, but safety and immunogenicity concerns have brought non-viral gene delivery to the forefront. Polymers are a popular non-viral vector choice due to their versatility, and both synthetically produced [26,27,28,29,30,31] and naturally occurring [32,33,34,35,36] polymers have shown success in nucleic acid delivery. In particular, chitosan is a natural polymer that has many pro-wound-healing attributes [37,38,39,40] and has been successfully used to deliver plasmids for regenerative purposes [41,42]. Thus, we hypothesized that delivery of a plasmid encoding human CA5-HIF-1α via chitosan particles would enhance angiogenesis and improve wound healing. Here, we report the formulation of chitosan particle/CA5-HIF-1α plasmid complexes and explore their angiogenic potential. We further investigate their use as a wound-healing therapeutic in an excisional wound-healing model in db/db mice with a particular emphasis on angiogenesis in newly healed tissue.

## 2. Results

### 2.1. Characterization of Chitosan Particle/HIF-1α-CA5 Plasmid Complexes

**Figure 1:**
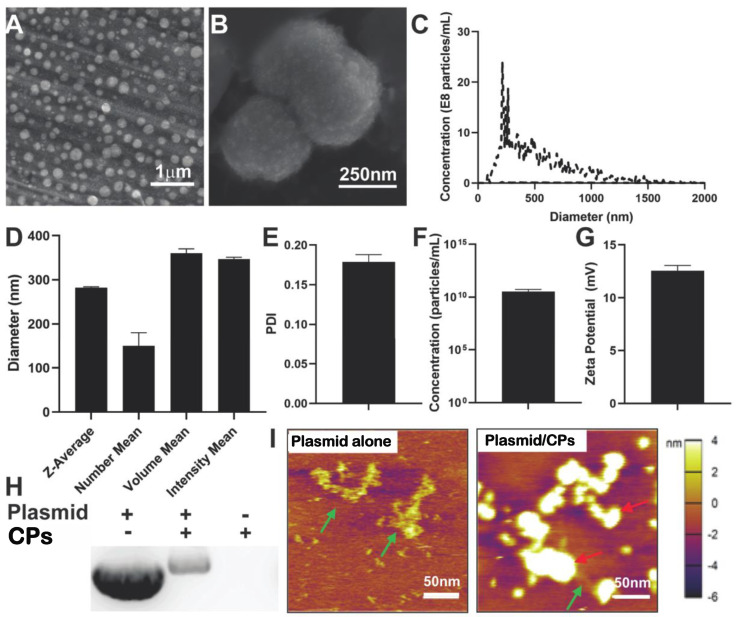
Characterization of chitosan particles (CPs) and chitosan particle/CA5-HIF-1α plasmid complexes (HIF/CP). Scanning electron microscopy images at (**A**) low magnification and (**B**) high magnification of particles. (**C**) Nanoparticle tracking analysis showing particle size distribution. (**D**) Dynamic light-scattering analysis of CPs showing z-average, number mean, volume mean, and intensity mean diameters. (**E**) Polydispersity index of CPs. (**F**) Concentration of CPs as determined by nanotracking analysis. (**G**) Zeta potential of CPs showing particles have a positive charge. (**H**) Gel electrophoresis of CA5-HIF-1α plasmids alone along with 50% CPs in solution with CA5-HIF-1α plasmid, and 50% CPs alone. (**I**) Atomic force microscopy images of CA5-HIF-1α plasmids alone and complexed with 50% CPs in solution (green arrows: plasmid DNA; red arrows: chitosan particles). For all panels, *n* = 3.

A common process for generating chitosan particles (CPs) used in drug and gene delivery studies is exemplified by the method of Calvo et al. [43]. Therapeutic cargo is added to a solution containing electrostatic crosslinker, typically tripolyphosphate, and becomes encapsulated when combined with chitosan solution. In this study, however, we first created a suspension of chitosan particles and then used them to form plasmid/particles complexes as a therapeutic formulation. Chitosan particles were synthesized using a high-molecular-weight chitosan polymer of 310–375 kDa with a degree of deacetylation greater than 80%, as described in the Materials and Methods. ^1^H NMR was performed on the chitosan polymer that was used in all subsequent studies to verify chemical structure with chitosan spectra found in the literature [44,45], and peaks unique to chitosan were identified on the spectra at 1.8 ppm and 3 ppm (Figure S1). Scanning electron microscopy (SEM) images showed the presence of nanoparticles of various sizes (Figure 1A). A highly resolved image revealed the topology of the spherical chitosan nanoparticles (Figure 1B). Nanoparticle tracking analysis of these particles measured a size distribution curve with a peak diameter of 265 nm (Figure 1C). Dynamic light-scattering analysis of the CPs was also performed to obtain a variety of weighted mean diameters (Figure 1D). The polydispersity index (PDI) was measured to be 0.179 ± 0.009 (Figure 1E). The zeta potential of the CPs was 12.57 ± 0.48 mV (Figure 1F). Fourier transform infrared spectroscopy (FTIR) of chitosan in a 1% acetic acid solution showed characteristic peaks for a N-H stretch between 3000 and 3200 nm and a carbonyl group between 1600 and 1700 nm (Figure S2). FTIR of chitosan nanoparticles—formed after the dropwise addition of tripolyphosphate—showed similar peaks for a N-H stretch and carbonyl group (Figure S2), indicating a physical, electrostatic crosslinking of chitosan versus chemical crosslinking in which the polymer is modified.

CA5-HIF-1α plasmids were used to create chitosan particle/CA5-HIF-1α plasmid complexes (HIF/CPs), which always contained 1 mg/mL plasmid DNA. Gel electrophoresis was used to separate plasmids that were not complexed with nanoparticles (Figure 1H). When compared to plasmid DNA without particles, plasmid complexes with particles showed a visible reduction in free plasmids. Chitosan particles alone were also used as a control for each group to ensure particles were not producing a signal found in the gel. A gradient of particles in solution was also performed from 0% to 87.5% and showed a reduction in free plasmids with increasing particle concentration (Figure S3). However, plasmid aggregation started to occur at a particle concentration of 87.5%. Atomic force microscopy was performed on naked CA5-HIF1α plasmid DNA as well as HIF/CPs. CPs were observed to be co-localized with plasmid DNA, potentially indicating a direct interaction (Figure 1I).

### 2.2. In Vitro Assessment of HIF/CPs Angiogenic Activity

**Figure 2:**
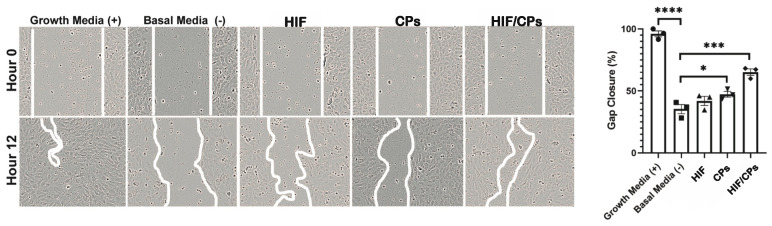
In vitro assessment of HIF/CPs’ angiogenic activity. HUVECs were treated with growth media only (EGM; positive control), basal media only (EBM; negative control), 100 μg/mL CA5-HIF-1α plasmids (HIF) in basal media, 50% chitosan particle solution (CPs) in basal media, or 100 μg/mL CA5-HIF-1α plasmids with 50% chitosan particles in solution (HIF/CPs) in basal media. Brightfield images were taken at 0 h and 12 h post-wounding of the cell layer. Representative images for each group and time point are displayed, and the gap area is outlined with a white line. Statistical significance was calculated using one-way ANOVA with Holm–Sidak’s multiple comparison test (* *p* < 0.05, *** *p* < 0.001, and **** *p* < 0.0001) (*n* = 3).

A scratch assay using HUVECs was used to determine the angiogenic potential of HIF/CPs. Three independent preparations were used in the assay. HUVECs were treated with 100 μg/mL (based on DNA content) CA5-HIF-1α plasmids with 50% chitosan particles (HIF/CPs) in basal media, and 50% chitosan particles alone (CPs) in basal media showed improved gap closure when compared to basal media. However, HIF/CPs had a higher and more statistically significant improvement in gap closure (65 ± 3% compared to 35 ± 4% for basal media; *p* = 0.0001) than CPs alone (47 ± 2% compared to 35 ± 4% for basal media; *p* < 0.05) (Figure 2).

### 2.3. Verification of In Vivo Transfection Using Luciferase Plasmid/NP Complexes

**Figure 3:**
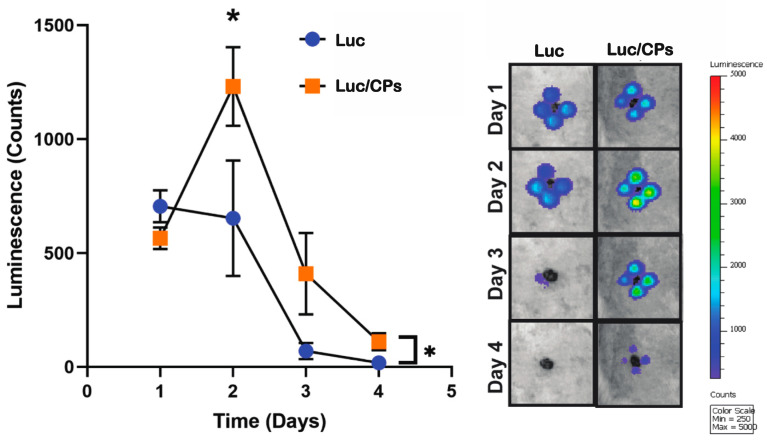
Luciferase expression over time when using chitosan particles for plasmid transfection. Verification of transfection efficiency of luciferase plasmids (Luc) using chitosan particles (CPs) in an in vivo reporter assay wound model. Statistical significance was calculated using two-way ANOVA with Holm–Sidak’s multiple comparison test (* *p* < 0.05) (*n* = 3).

An in vivo luciferase reporter assay was performed to assess the transfection efficiency of the CPs. Two 8 mm wounds, with each at least 5 cm apart across the midline to account for anatomical differences, were created on the dorsum of Sprague Dawley rats. Immediately after wounding, the wounds on each rat received either 50 μg of luciferase-encoded plasmids with 50% chitosan nanoparticles (Luc/NPs) or 50 μg of luciferase plasmids alone (Luc). Luciferin was administered to each rat intraperitoneally on days 1, 2, 3, and 4 post-wounding to monitor plasmid expression. Luc/NPs showed an overall statistically significant improvement in luciferase expression during the 4-day period when compared to Luc (*p* < 0.05). Additionally, maximum expression was observed on day 2, with 1200 ± 200 luminescence counts for Luc/NPs, which is significantly higher than the 600 ± 300 luminescence counts found for Luc (*p* < 0.05) (Figure 3).

### 2.4. In Vivo Tissue and Molecular Analysis of HIF/CPs

**Figure 4:**
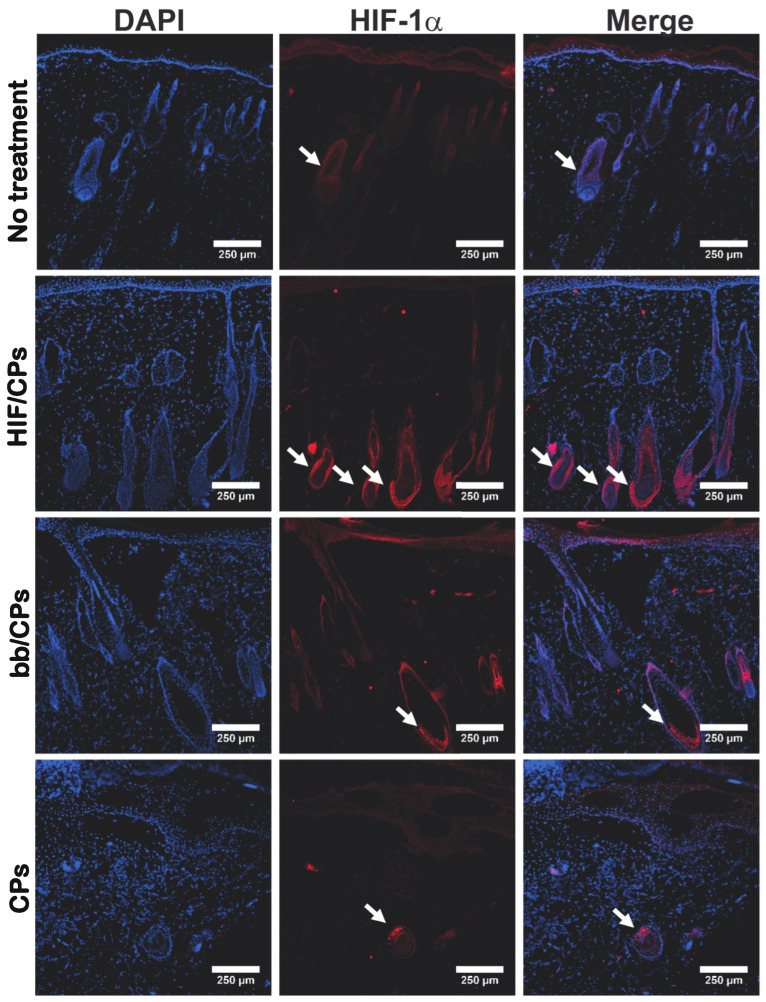
HIF/CPs induce expression of HIF-1α among hair follicles on wound edge in Sprague Dawley rats. Immunohistochemistry of HIF-1α in skin surrounding the wound with no treatment, 50 μg (based on plasmid content) of 50% chitosan particle/CA5-HIF-1α plasmid complexes (HIF/CPs), 50 μg (based on plasmid content) of 50% chitosan particles with plasmid backbone (bb/CPs), or 50% chitosan particles (CPs). Treatments were injected into the dermis of skin surrounding the wound on day 3 post-wounding, and samples were collected on day 5 for analysis via immunohistochemistry (hair follicular regions expressing HIF-1α shown with white arrows).

**Figure 5:**
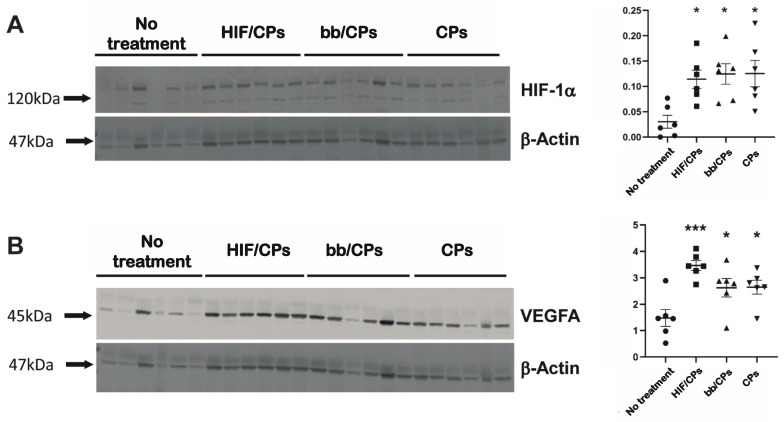
Western blots of skin treated with HIF/CPs in Sprague Dawley rats for HIF-1α and VEGFA. Western blots for (**A**) HIF1α and (**B**) VEGFA of skin with no treatment or treatment with 50 μg (based on plasmid content) of 50% chitosan particle/CA5-HIF-1α plasmid complexes (HIF/CPs), 50 μg (based on plasmid content) of 50% chitosan particles with plasmid backbone (bb/CPs), or 50% chitosan particles (CPs). Treatments were injected into the dermis of skin surrounding the wound on day 3 post-wounding, and samples were collected on day 5 for analysis via Western blot. Relative expression of HIF1α and VEGFA from each sample was determined via normalization to β-actin from its respective lane. Statistical significance was calculated using one-way ANOVA with Holm–Sidak’s multiple comparison test (* *p* < 0.05, *** *p* < 0.001) (*n* = 6).

Healthy, retired breeder Sprague Dawley rats were used to investigate the expression of HIF-1α as well as VEGFA, a well-established protein downstream of HIF-1α [46,47]. Two days post-treatment, in accordance with the luciferase data in Figure 3, tissue was harvested for molecular analysis during peak protein expression. Staining tissue sections for HIF-1α showed HIF/CPs induced the densest expression of HIF-1α among hair follicles among all groups examined (Figure 4). A similar intensity but less dense visual expression of HIF-1α among hair follicles was also seen in both groups containing chitosan particles (bb/CPs and CPs), while the no-treatment control showed the lowest intensity and lowest density of HIF-1α expression among hair follicles. HIF/CPs, bb/CPs, and CPs all had statistically significant higher levels of HIF-1α and VEGFA when compared to the no-treatment control via Western blot (Figure 5).

### 2.5. In Vivo Excisional db/db Wound-Healing Model and Histological Analysis for Angiogenesis

**Figure 6:**
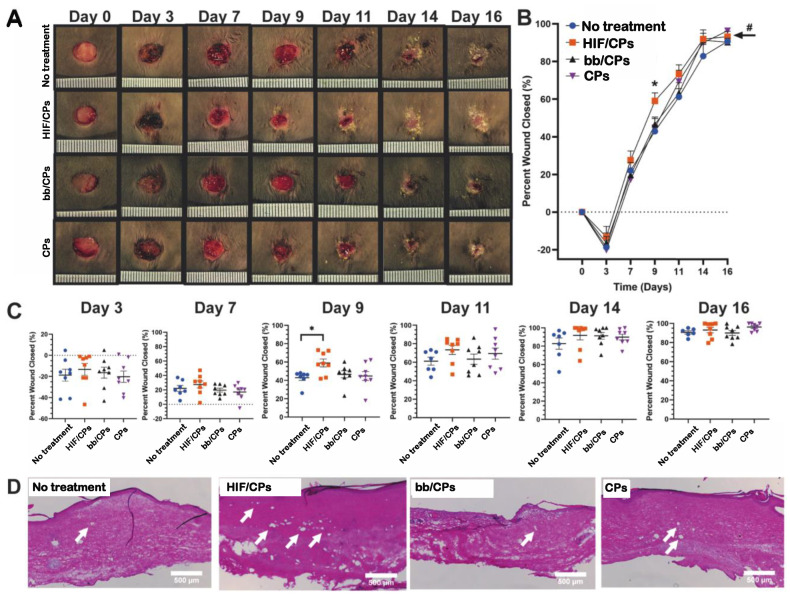
HIF/CPs improve wound healing in an excisional db/db mouse model. (**A**) Representative images of 8 mm wounds with no treatment or treatment with HIF/CPs, bb/CPs, or CPs. (**B**) Wound closure over time and (**C**) individual wound sizes on each day of measurements as assessed using digital planimetry. (**D**) Representative H&E histology of healed tissue from day 21. White arrows indicate blood vessels within the tissue. In the no-treatment group, one mouse perished by day 7 and another was sacrificed on day 14 due to wound infection. # indicates statistical significance (*p* < 0.01) of improved overall healing, assessed using a two-way ANOVA of HIF/CPs compared to both CPs and no treatment. Statistical significance was calculated using (**B**) two-way and (**C**) one-way ANOVA with Holm–Sidak’s multiple comparison test (* *p* < 0.05) (*n* = 8).

**Figure 7:**
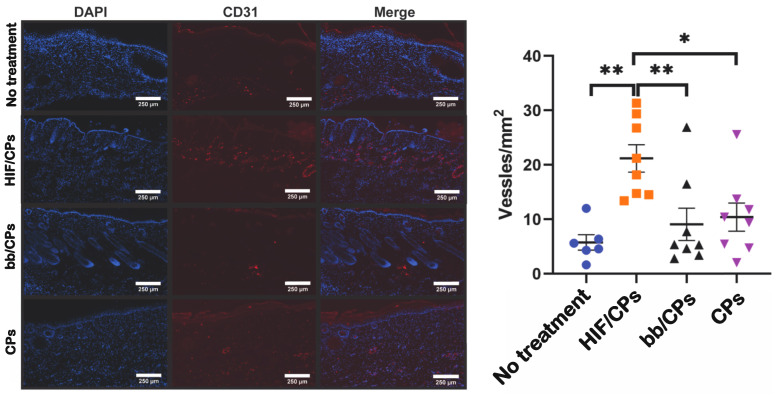
HIF/CPs induce angiogenesis in healed tissue of db/db mice. CD31+ immunohistochemistry in day 21 tissue from db/db mice. The number of positive structures was counted and tissue in field of view was measured in vessels/mm^2^. The tissue of all 8 mice per group was analyzed, except for the 2 mice in the no-treatment group, which had either perished or were sacrificed prematurely due to wound infection. Statistical significance was calculated using one-way ANOVA with Holm–Sidak’s multiple comparison test (* *p* < 0.05, ** *p* < 0.01; *n* = 8).

Having confirmed that chitosan particles showed angiogenic activity in vitro and increased levels of HIF-1α and VEGFA in vivo, and that in vitro angiogenic activity and VEGFA levels were further increased with the addition of the CA5-HIF-1α plasmid, we then assessed HIF/CPs treatment in an excisional wound-healing model in db/db mice. Experimental groups were the same as before, with HIF/CPs, bb/CPs, CPs, and the no-treatment control. One animal from the no-treatment group perished before day 7 and another animal from the no-treatment group was sacrificed on day 14 due to a wound infection. HIF/CPs induced a statistically significant improvement in healing overall when compared to both the no-treatment and CP treatment groups assessed with two-way ANOVA (Figure 6B; *p* < 0.01), whereas both bb/CPs and CPs alone did not induce any significant changes when compared to no treatment. Additionally, the data show that HIF/CPs may increase the rate of wound closure, as observed by the significantly increased wound closure at day 9 (Figure 6B,C; 59 ± 4% compared to 43 ± 3% for no-treatment control, *p* < 0.05). To assess angiogenesis, the tissue of all mice remaining in each group was harvested on day 21 for H&E staining (Figure 6D) and CD31 immunohistochemistry (Figure 7). Animals treated with HIF/CPs had significantly more CD31+ vessels (21 ± 3 vessels/mm^2^) when compared to those not treated (6 ± 1 vessels/mm^2^; *p* < 0.01), bb/CPs (9 ± 3 vessels/mm^2^; *p* < 0.01) and those treated with CPs (10 ± 3 vessels/mm^2^; *p* < 0.05) (Figure 7). There was no statistically significant difference among the no-treatment, bb/CP, and CP groups.

## 3. Discussion

Gene delivery for wound healing has been an active area of research for our group and others [25,41,42], and non-viral methods of gene delivery have particularly been of interest due to the reduction in carcinogenesis, immunogenicity, and cost when compared to viral vectors [48]. Although transduction using viral vectors is much more efficient for gene delivery, polymeric formulations have also shown success [26,27,28,29,32,33,34,35,36]. In this study, we examined the potential of utilizing the pro-wound-healing polymer chitosan for the delivery of a DNA plasmid encoding human CA5-HIF-1α, in which amino acids 392–520 are deleted to prevent O_2_-dependent degradation by prolyl hydroxylases [24], to improve angiogenesis and enhance wound healing.

The data show that chitosan can be formulated as particles with the addition of the ionic crosslinker tripolyphosphate (Figure 1A–G), and that these particles can be used to create complexes with plasmid DNA (HIF/CPs; Figure 1H,I). Additionally, the number of free plasmids remaining in the solution is reduced with increasing concentrations of particles (Figure S3). However, DNA aggregation results if the particle concentration is too high. A study by Gomes et al. showed the possibility of tailored gene expression due to the tunable enzymatic degradation of different chitosan polymers and removal from DNA plasmids [36]. The particle/plasmid complexes developed here may also be able to achieve similar results due to the ability to vary particle concentration in solutions with plasmid DNA, with lower concentrations potentially resulting in higher peaks and faster expression, as opposed to higher concentrations resulting in blunted peaks but extended expression. To determine the angiogenic activity of HIF/CPs, we utilized an in vitro angiogenic assay to assess the activity induced by our complexes. We found that CPs alone had moderate activity in this assay, but this activity was significantly increased with HIF complexation (Figure 2). Similar molecular findings arose in vivo, with all groups containing chitosan particles showing comparable levels of increased HIF-1α to the no-treatment group; however, HIF/CPs showed the most significantly increased levels of VEGFA compared to the plasmid control (bb/CPs) and vector control (CPs), indicating an active role of the human CA5-HIF-1α-encoded plasmid. Overall, the results suggest that increased HIF-1α potency, rather than total level of HIF-1α, is responsible for the therapeutic effects observed. Additionally, HIF-1α expression was most intensely observed within hair follicles, suggesting hair follicle stem cells may be chiefly responsible for the expression of HIF-1α from the delivered plasmid.

Chitosan has been used to deliver various therapeutics to the skin for wound-healing purposes [41,42,49,50,51,52]. A subset of these studies have specifically utilized chitosan for gene delivery [41,42]. Most notably, Lord et al. delivered DNA plasmids to the skin for the expression of a pro-angiogenic proteoglycan and protein. They created chitosan scaffolds containing a plasmid encoding perlecan and VEGF for use in full-thickness dermal wound-healing models and found that their treatment was able to improve healing in both normal and diabetic rats [41]. In this study, intradermal delivery of HIF/NCs was able to improve wound healing in an excisional, full-thickness wound in db/db mice. Additionally, we found that HIF/CPs induced a significantly increased number of CD31+ vessel structures in healed tissue compared to bb/CPs, CPs, and no treatment.

Several limitations to our study must also be acknowledged. Chitosan particle concentrations of less than 87.5% did not aggregate plasmid DNA and thus are also capable of intradermal delivery. These other concentrations may produce more optimized expression profiles of HIF-1α for wound healing. We also only tested one degree of deacetylation of chitosan. Other forms of chitosan have also been successful in enhancing transfection [53,54] and thus may offer other forms of expression. We did not examine different chitosan particle formulations, nor look extensively at particle stability to determine potential effects on DNA plasmid complexation and delivery. We also did not see a difference in HIF-1α protein levels from HIF/CPs (Figure 5A) when compared to the other chitosan particle groups, although the HIF/CPs did produce the most statistically significant increase in VEGFA (Figure 5B), a statistically significant improvement in wound healing (Figure 6), and a statistically significant increase in the number of vessel structures in healed tissue (Figure 7), which was not observed among other groups containing chitosan particles. Designing two sets of primers for RT-qPCR specific to only human or rat HIF-1α mRNA would lead to different expression levels of human and rat HIF-1α mRNA, which could alleviate a potential convoluting factor. We also recognize that the mouse model employed here varies significantly from human wound-healing physiology. Lastly, hair follicle stem cells actively proliferate [55,56]. As this was the cell type that predominately displayed expression of HIF-1α, their proliferating nature may have assisted in the transfection efficiency observed for HIF/CPs. This may have been necessary for the therapeutic efficacy of the treatment and may not be carried over in treatments involving non-dividing cells.

In summary, we report the potential of using chitosan particles for the delivery of a DNA plasmid encoding human CA5-HIF-1α to induce increased angiogenesis and improved cutaneous wound healing. While a single gene was investigated here, the use of this formulation for the delivery of plasmid DNA to the skin may be broadly applicable to many pro-wound-healing genes. Utilizing a non-viral gene delivery vector that has proven pro-wound-healing capabilities is beneficial in developing successful wound therapies for clinical use. Optimization of the specific chitosan polymer used as well as the concentration of formulated particles may help promote appropriate expression of key angiogenic genes for wound healing and other procedures requiring revascularization.

## 4. Materials and Methods

### 4.1. Plasmid Preparation

Plasmid DNA encoding the human CA5-HIF-1α gene and firefly luciferase gene was provided by Nature Technology Corporation (NTC) (Lincoln, NE, USA). pCMV-GFP was a gift from Connie Cepko (Addgene plasmid #11153; http://n2t.net/addgene:11153; RRID:Addgene_11153 (accessed on 12 July 2021). To obtain a pCMV-CA5-HIF-1α plasmid (CA5-HIF-1α plasmid), CA5-HIF-1α from NTC-CA5-HIF-1α was amplified using KOD DNA Polymerase (EMD Millipore, Burlington, MA, USA; 71086-3), and the plasmid backbone from pCMV-GFP was also amplified using KOD Polymerase. CA5-HIF-1α was then inserted into pCMV backbone using a Gibson Assembly Cloning Kit (New England BioLabs, Ipswich, MA, USA; E5510S). A plasmid backbone was formed from pCMV-GFP via backbone amplification using KOD polymerase and the Gibson Assembly Cloning Kit. The final CA5-HIF-1α plasmid and plasmid backbone were sent for Sanger sequencing to Genewiz to verify the appropriate sequence. Maxiprep Kits (Qiagen, Hilden, Germany; 12162) were used to amplify the plasmids for use in subsequent studies. Isolated plasmids were stored in saline at −20 °C.

### 4.2. Preparation and Characterization of Chitosan Particle/CA5-HIF-1α Plasmid Complexes

Chitosan particles were created using the ionic gelation method, as previously described [43]. Briefly, a 50 mL solution containing 0.125 g of sodium tripolyphosphate (Sigma Aldrich, St. Louis, MO, USA; 238503) was added dropwise to a 50 mL solution of 0.125 g of chitosan (310–375 kDa, >80% DDA) (Sigma Aldrich, St. Louis, MO, USA; 419419) in acetic acid (Sigma Aldrich, St. Louis, MO, USA; 695092) that was diluted to 1%. All solutions were prepared using saline (VWR International, Radnor, PA, USA). During the titration process, constant stirring was achieved using a magnetic stir bar. The two solutions were completely combined after an hour and the chitosan particles were continually stirred for an additional 30 min. The solution was separated into two, 50 mL conical tubes and centrifuged for 10 min at 2630× *g* and 4 °C in an Eppendorf^TM^ Centrifuge 5810R (Eppendorf, Hauppauge, NY, USA). The supernatant was collected and centrifuged a second time for 5 min in 1 mL Eppendorf tubes at 4620× *g* at room temperature in a Costar^®^ Centrifuge (Corning, Corning, NY, USA). The supernatant was collected as a stock 100% particle suspension. The order of addition in creating chitosan particle/plasmid complexes was saline, particle suspension, and then 1 mg/mL plasmid DNA. The number of particles was determined as the percent of stock solution in the final volume with 1 mg/mL plasmid DNA.

A Bruker AVIII 600 MHz NMR was used to perform 1D ^1^H NMR on the chitosan polymer. A Vertex 70 Fourier Transform Infrared Spectroscope (Bruker, Billerica, MA, USA) was used to detect functional groups characteristic of the chitosan polymer before and after the addition of the tripolyphosphate crosslinker. A Malvern ZetaSizer ZS90 (Malvern Instrument Ltd., Malvern, UK) was used to measure the zeta potential of the chitosan particles. Nanoparticle tracking analysis using a Malvern NanoSight LM10 (Malvern Instrument Ltd., Malvern, UK) was carried out to measure the size distribution and total concentration of chitosan particles in the suspension. Dynamic light-scattering analysis of chitosan particles was performed using a Malvern ZetaSizer ZS90 (Malvern Instrument Ltd., Malvern, UK) to measure the polydispersity index (PDI) along with the z-average diameter, number mean diameter, volume mean diameter, and intensity mean diameter of the particles.

Scanning electron microscopy (SEM) using a Hitachi SU-70 Scanning Electron Microscope (Hitachi, Tokyo, Japan) was used to verify the morphology of chitosan particles. Briefly, the sample was deposited at a volume of 5 µL onto an aluminum imaging mount (Electron Microscopy Sciences, Hatfield, PA, USA) and placed in a desiccator with a vacuum seal until the solution was completely evaporated for imaging. Gel electrophoresis was used to assess free DNA left in solution with varying concentrations of particles. Solutions contained 1 mg/mL plasmid DNA and either 0%, 1%, 5%, 10%, 25%, 50%, or 87.5% stock particle suspensions in the final volume. Briefly, 5 μL from each of the complexes and 1 μL of 6× gel loading dye (New England BioLabs, Ipswich, MA, USA) were run through a 1% agarose gel (Thermoscientific, Waltham, MA, USA; R2801) containing 0.02% Gelred (Biotium, Fremont, CA, USA; 41003) for approximately 1 h at 120 V. Additionally, both 5% and 50% particle solutions with and without DNA were also run through the gel using the same parameters. Atomic force microscopy (AFM) was used to image chitosan particle/CA5-HIF-1α plasmid complexes at different concentrations. An MFP-3D Atomic Force Microscope (Asylum Research, Santa Barbara, CA, USA) was used to image the in situ complexation between the particles and plasmid DNA at particle concentrations of 0%, 1%, 5%, 10%, and 50%. Briefly, 70 µL of a 1 mM NiCl_2_ solution was deposited onto a mica imaging substrate (Ted Pella, Redding, CA, USA) for 5 min and then removed. Then, 70 µL of a 20 µg/mL solution of pDNA was deposited onto the surface for 30 min before removing the solution and washing it three times with saline. The particle-containing solution was diluted to 1%, 5%, 10%, or 50% then added to the surface for 30 min before washing it three times with saline. Finally, 70 µL of saline was added to image the complexes in aqueous conditions.

### 4.3. In Vitro Assessment of Angiogenic Activity

An in vitro scratch assay using human umbilical vein endothelial cells (HUVECs) was used to test the angiogenic properties of chitosan particle/CA5-HIF-1α plasmid complexes. A final concentration of 50% particles was chosen for use in solution with 1 mg/mL plasmid DNA for all subsequent studies and is referred to as HIF/CPs. P5 HUVECs were seeded at 200,000 cells/well in gelatin-coated 24-well plates with 500 μL total volume/well in EGM2 and allowed to grow until a uniform monolayer was formed (24 h). The medium was replaced with an endothelial basal medium (EBM2) (Promocell, Heidelberg, Germany; C-22221) supplemented with 0.5% FBS for 24 h to serum-starve the cells. The cell monolayer was then scratched using a 200 μL pipette tip (Rainin, Columbus, OH, USA; 30389243). The media were then replaced with the same low-serum media. Following this, 100 μg/mL (based on DNA content) HIF/CPs, 100 μg/mL CA5-HIF-1α plasmids alone, or 50% CPs alone was added to the well. EGM-2 and EBM-2 were used as positive and negative controls, respectively. The cell gap was imaged at 0 h and 12 h. Overall gap closure was determined as the percentage of area covered by endothelial cells versus the gap remaining after 12 h using ImageJ version 1.53t, as previously described [57].

### 4.4. In Vivo Luciferase Reporter Assay Using Chitosan Particles in Sprague Dawley Rats

Transfection efficiency of the chitosan particles using an in vivo luciferase assay with a plasmid encoding firefly luciferase (NTC, Lincoln, NE, USA) was performed in Sprague Dawley rats. An excisional wound model utilizing six retired breeder Sprague Dawley rats (400–600 g) from Charles River (Wilmington, MA, USA) was employed. All procedures involving rats were approved by the Johns Hopkins University Animal Care and Use Committee. Rats were anesthetized with 3% isoflurane (Baxter Healthcare Corporation, Deerfield, IL, USA) and had their entire dorsum shaved. Two 8 mm punch biopsies (Integra, Plainsboro, NJ, USA) were performed on the dorsum of each rat at least 5 cm apart along the midline. Buprenorphine (0.05 mg/kg) was subcutaneously administered on days 0, 1, and 2 for pain. Treatments were administered only once during the experiment, and on day 0, post-wounding was carried out. Specifically, four 50 μL injections equidistant around the wound were performed. Treatments included 50 μg of luciferase plasmids with 50% chitosan particles (Luc/CPs) or 50 μg of luciferase plasmids alone (Luc).

An IVIS Xenogen Camera (Caliper Life Science, Alameda, CA, USA) was used to obtain photographs of rats superimposed with luminescence images on days 1, 2, 3, and 4 post-injection. Prior to imaging, on each day, rats were anesthetized with 3% isofluorane and given 5 mL intraperitoneal injections of 15 mg/mL luciferin (Biosynth International, Itasca, IL, USA). Thirty minutes following injections, the rats were imaged. Luminescence (measured in counts) was calculated using the Living Image 4.5.2 (PerkinElmer, Waltham, MA, USA) software for equal-sized regions of interest at each injection site. All injections per rat were averaged, and average luminescence was used for statistical comparison.

### 4.5. In Vivo Molecular Study of HIF/CPs in Sprague Dawley Rats

An excisional wound model utilizing six retired breeder Sprague Dawley rats (400–600 g) from Charles River (Wilmington, MA, USA) was employed. All procedures involving rats were approved by the Johns Hopkins University Animal Care and Use Committee, and all procedures followed the Johns Hopkins University ACUC. Rats were anesthetized with 3% isoflurane (Baxter Healthcare Corporation, Deerfield, IL, USA) and had their entire dorsum shaved. Four 8 mm punch biopsies (Integra, Plainsboro, NJ, USA) were performed on the dorsum of each rat. Wounds were administered in a square pattern, with each at least 5 cm apart. Buprenorphine (0.05 mg/kg) was subcutaneously administered on days 0, 1, and 2 for pain. Treatments were administered via four equidistant 50 μL intradermal injections around the wound once during the experiment, and on day 3, post-wounding was carried out. Treatments included 50 μg of CA5-HIF-1α plasmids with 50% chitosan particles (HIF-CA5/CPs), 50 μg of plasmid backbone with 50% chitosan particles (backbone/CPs), 50 μg of chitosan particles alone (CPs), or no treatment controls.

Tissue was harvested on day 5 for molecular analysis. Briefly, a semicircle region of the caudal end of each wound was collected for immunohistochemistry and placed in a cryomold (VWR International, Radnor, PA, USA; 4557) and covered with OCT medium (Leica Biosystems, Deer Park, IL, USA; 3801480). The tissue was positioned so that the cut edge was sitting at the bottom of the mold. The cassette was gently transferred onto a metal surface cooled with dry ice and ethanol until the OCT medium solidified. Tissue was stored at −80 °C for no longer than 1 week before sectioning. ACM1950 Cryostat (Leica Biosystems, Deer Park, IL, USA) was used to section 10 μm of tissue. Tissue was fixed and permeabilized in a 1:1 methanol/acetone solution at −20 °C.

Immunohistochemistry was performed for HIF-1α. Briefly, sections were washed with tris-buffered saline (TBS) for 2 min, pre-blocked in 1% Bovine Serum Albumin (Sigma Aldrich, St. Louis, MO, USA; A2058)/5% Donkey Serum (Sigma Aldrich, D9663) in TBS for 30 min, incubated with rabbit polyclonal HIF-1α antibody (Abcam, Cambridge, UK; 216842) at 1:100 in blocking solution for 60 min at room temperature, washed with TBS two times for 5 min each, incubated with Alexa Flour 647 donkey anti-rabbit secondary antibody (Invitrogen, Waltham, MA, USA; A31573) for 60 min at room temperature shielded from light, and washed with TBS two times for 5 min each. Vectashield Antifade Mounting Media (Vector Laboratories, Newark, CA, USA; H-1200) containing DAPI were added to a No. 1.5 micro coverslip (VWR International, Radnor, PA, USA; 48393-195) and placed over the tissue section. Clear fingernail polish was used to seal the coverslip to the slide. Fluorescent images for DAPI and Cy5 were obtained using an Eclipse Ti2 Microscope (Nikon, Minato City, Tokyo, Japan) at 10× magnification.

A 5 mm punch biopsy was used to sample an injected region at the cephalic end of each wound for protein isolation and immediately homogenized in 200 μL of Pierce^TM^ RIPA Buffer (Thermoscientific, Waltham, MA, USA; 89901) supplemented with 1% Halt^TM^ Protease Inhibitor Cocktail (Thermoscientific, Waltham, MA, USA; 1861278) and 1% 0.5 M EDTA Solution (Thermoscientific, Waltham, MA, USA; 1861275). Western blots were performed for HIF-1α and VEGFA. Briefly, total protein content was measured using a BCA assay (Biosciences, 786-571). A total of 50 μg of protein per well was loaded into a 26-well, 4–15% precast polyacrylamide gel (BioRad, Hercules, CA, USA; 5671085) and transferred onto a 0.2 μm nitrocellulose membrane (BioRad, Hercules, CA, USA; 1704159). The membrane was briefly washed in 1× PBS, and then a 1:1 solution of 1× PBS:Intercept^®^ Blocking Buffer (Li-Cor, 927-70001) was used to pre-block the membrane for 1 h at room temperature. Then, the membrane was incubated overnight under rocking conditions at 4 °C in a 1:1 solution of 1× PBS with 0.1% Tween:Intercept^®^ Blocking Buffer with 1:1000 of either rabbit polyclonal HIF-1α (Abcam, Cambridge, UK; 216842) or rabbit polyclonal VEGFA (Abcam, Cambridge, UK; 46154) antibody along with 1:2000 mouse monoclonal β-actin (Abcam, Cambridge, UK; 8226) antibody. The membrane was then washed 3× with 1× PBS with 0.1% Tween. The membrane was incubated for 1 h at room temperature with fluorescent goat secondary antibodies for both mouse (A680; Licor, Lincoln, NE, USA; 926-68070) and rabbit (A800; Licor, Lincoln, NE, USA; 926-32211) at a 1:10,000 dilution. The membrane was washed again 3× with 1× PBS with 0.1% Tween. A Li-Cor Odyssey CLx was used to image the membrane. HIF-1α and VEGFA relative expression levels in each lane were standardized to the respective β-actin stain on the same blot using the densitometry feature of Image Studio (Version 5.0) software. Relative expression levels standardized to β-actin were used for statistical comparisons.

### 4.6. In Vivo Functional Study of HIF/CPs in db/db Mice

An impaired wound-healing animal model utilizing db/db mice (40–50 g) from Jackson Laboratory (Bar Harbor, ME, USA) was used. All procedures involving mice were approved by the Johns Hopkins University Animal Care and Use Committee. Mice were anesthetized with 1.5% isoflurane (Baxter Healthcare Corporation, Deerfield, IL, USA) and had their entire dorsum shaved. One 8 mm punch biopsy (Integra, Plainsboro, NJ, USA) was performed on the dorsum of each mouse. Buprenorphine (0.05 mg/kg) was subcutaneously administered on days 0, 1, and 2 for pain. Wounds were matched based on day 0 wound size. A total of 24 mice were used (8 per group). Treatments were administered only once during the experiment on day 3 post-wounding. As above, four 50 μL injections equidistant around the wound were administered. Treatments included 50 μg of HIF/CPs, 50 μg of bb/CPs, 50 μg of CPs, or no-treatment controls. Photographs with a ruler showing the size in millimeters and tracings of the wound were taken on days 0, 3, 7, 9, 11, 14, and 16, and wound size was measured using digital planimetry. Wound size was determined as the percentage of area of the wound to the wound size on day 0.

Healed tissues were biopsied on day 21 using a 12 mm punch. The tissue was cut down the center of the wound, and the same method as described above for HIF-1α immunohistochemistry was repeated to obtain 10 μm sections of tissue. To assess general tissue architecture, tissue sections were stained with H&E using the following protocol: deionized water wash for 2 min, hematoxylin (VWR International, Radnor, PA, USA; 75810-352) for 3 min, deionized water wash for 1 min, differentiator in 4% HCl in 95% ethanol for 1 min, deionized water wash for 1 min, bluing for 1 min in 1% NaCO_3_, deionized water wash for 1 min, 95% ethanol for 1 min, eosin (VWR International, Radnor, PA, USA; 75810-354) for 45 s, 95% ethanol for 1 min, 100% ethanol two times for 1 min each, and xylene two times for 2 min each. Sub-X Mounting Medium (Leica Biosystems, Deer Park, IL, USA; 3801740) was added to a No. 1.5 micro coverslip (VWR International, Radnor, PA, USA; 48393-195) and placed over the tissue section. Clear fingernail polish was used to seal the coverslip to the slide. Brightfield images were obtained using an Eclipse Ti2 Microscope (Nikon, Minato City, Tokyo, Japan) at 10× magnification. Additionally, tissue sections underwent CD31 staining to identify newly formed blood vessels using the same protocol described above for HIF-1α staining. In this instance, CD31 primary antibody (Abcam, Cambridge, UK; 28364) at 1:50 was used. Fluorescent images for DAPI and Cy5 were obtained using an Eclipse Ti2 Microscope (Nikon, Minato City, Tokyo, Japan) at 10× magnification. The numbers of vessels were counted and recorded, and the area of tissue was quantified using ImageJ. The number of vessels/mm^2^ of tissue was recorded for statistical analysis.

### 4.7. Statistical Analysis

Data are presented as mean ± SEM. Statistical significance was determined using either two-way ANOVA with Holm–Sidak’s multiple comparison test or one-way ANOVA with Holm–Sidak’s multiple comparison test. A *p*-value of less than 0.05 was considered significant. All statistical analyses were performed with Prism 7 (GraphPad Software, La Jolla, CA, USA). Notations for significance in figures are as follows: ns = *p* > 0.05, * = *p* < 0.05; ** = *p* < 0.01; *** = *p* < 0.001; **** = *p* < 0.0001.

## Data Availability

Raw data is available from the authors upon reasonable request.

## Supplementary Materials

The following supporting information can be downloaded at: https://www.mdpi.com/article/10.3390/ijms241814095/s1.

## Abbreviations

HIF-1α: hypoxia-inducible factor 1-α; SEM, scanning electron microscopy; AFM, atomic for microscopy; FTIR, Fourier transform infrared spectroscopy; NPs, nanoparticles; HUVECs, human umbilical vein endothelial cells.
