# Supplementary material for: Chitosan Particles Complexed with CA5-HIF-1α Plasmids Increase Angiogenesis and Improve Wound Healing
Source: Int J Mol Sci. 2023 Sep 14;24(18):14095. doi: 10.3390/ijms241814095 (PMC10531456; doi:10.3390/ijms241814095)
Supplement: Supplementary file 1 [file ijms-24-14095-s001.zip › ijms-2592661-supplementary.pdf]

Supporting Information

Chitosan Particles Complexed with CA5-HIF-1a Plasmids Increase Angiogenesis and Improve Wound Healing

Louis J. Born[a,b], Sameer Bengali[b], Angela Ting Wei Hsu[b], Sanaz Nourmohammadi Abadchi[b], Kai-Hua Chang[b], Frank Lay[b], Aerielle Matsangos[b], Christopher Johnson[b], Steven M. Jay[a,c], John W. Harmon[b]

Figure S1

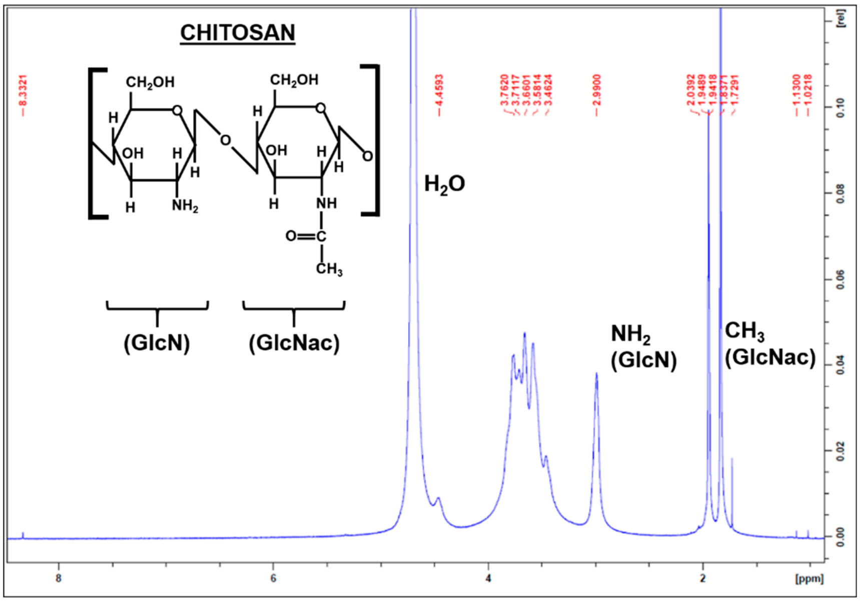

Figure S1: ^{1}H NMR spectroscopy of chitosan. Verification of chemical structure of chitosan using proton NMR. Peaks produced by hydrogens within GlcN unit and GlcNac unit are identified.

Figure S2

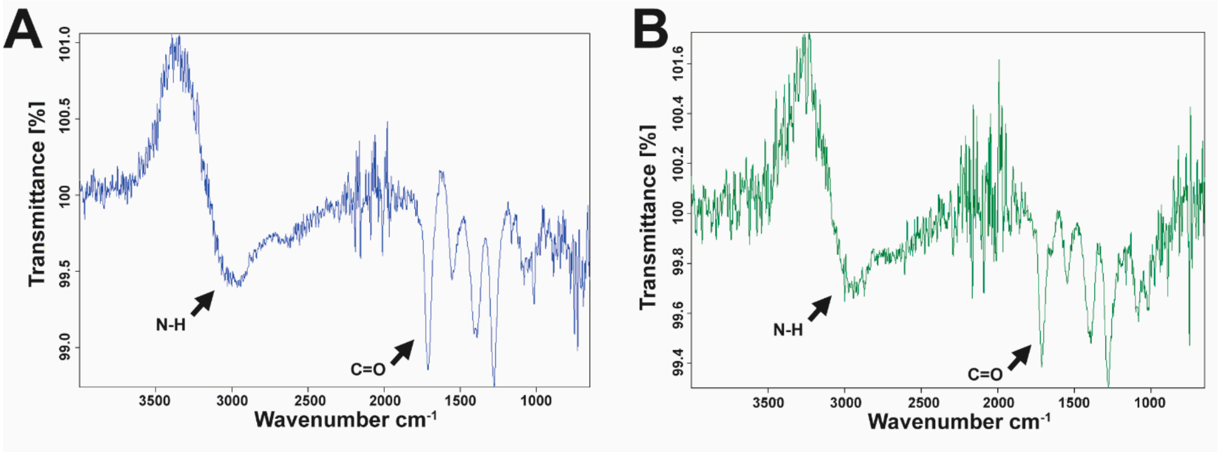

Figure S2: Fourier-transform infrared spectroscopy chitosan polymer and chitosan particles. FT-IR of chitosan polymer alone (A) and after addition of ionic crosslinker tripolyphosphate (B) (arrows show representative chemical properties of chitosan polymer).

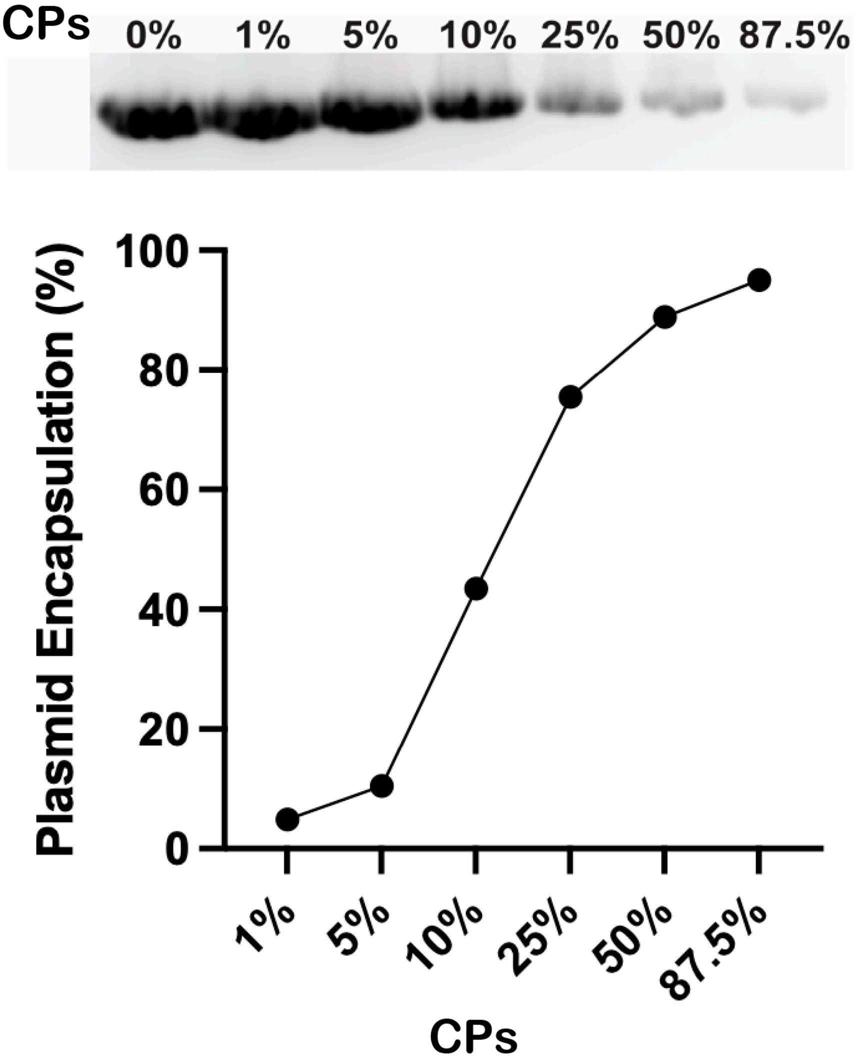

Figure S3. Increasing concentration of chitosan particles in solution with plasmid DNA. Increasing the amount of chitosan particles (CPs) in solution with the same amount of plasmid DNA decreases the amount of free plasmid left in solution. However, increased plasmid aggregation was visibly observed at concentrations of 87.5% particles in solution with plasmid.
